# Exosomes overexpressing miR-34c inhibit malignant behavior and reverse the radioresistance of nasopharyngeal carcinoma

**DOI:** 10.1186/s12967-019-02203-z

**Published:** 2020-01-08

**Authors:** Fang-Zhu Wan, Kai-Hua Chen, Yong-Chu Sun, Xi-Chan Chen, Ren-Ba Liang, Li Chen, Xiao-Dong Zhu

**Affiliations:** 1 Department of Radiation Oncology, Guangxi Medical University Cancer Hospital, Cancer Institute of Guangxi Zhuang Autonomous Region, Nanning, Guangxi People’s Republic of China; 2 Guangxi Key Laboratory of Early Prevention and Treatment for Regional High Frequency Tumor, Guangxi Medical University, Nanning, Guangxi People’s Republic of China; 3 Department of Oncology, Affiliated Wuming Hospital of Guangxi Medical University, Nanning, Guangxi People’s Republic of China

**Keywords:** microRNA-34c, β-Catenin, Nasopharyngeal carcinoma, EMT, Radioresistance

## Abstract

**Background:**

Malignant behavior and radioresistance, which severely limits the efficacy of radiation therapy (RT) in nasopharyngeal carcinoma (NPC), are associated with tumor progression and poor prognosis. Mesenchymal stem cells (MSCs) are used as a therapeutic tool in a variety of tumors. The aim of this study was to reveal the effect of tumor suppressor microRNA-34c-5p (miR-34c) on NPC development and radioresistance, as well as to confirm that exosomes derived from MSCs overexpressing miR-34c restore the sensitivity to radiotherapy in NPCs.

**Methods:**

Potentially active microRNAs were screened by cell sequencing, Gene Expression Omnibus (GEO) database analysis, and analysis of clinical serum samples from 70 patients. The expression of genes and proteins was detected by Western blotting, quantitative reverse transcription PCR (qRT-PCR), and immunohistochemistry (IHC). Proliferation, apoptosis, invasion, migration and radioresistance of NPC were detected. Luciferase reporter assays were used to verify the interactions of microRNAs with their downstream targets. MSCs exosomes were isolated by ultrafiltration and verified by electron microscopy and nanoparticle tracking technology.

**Results:**

The expression of miR-34c was associated with the occurrence and radiation resistance of NPC. In vitro and in vivo experiments indicated that overexpression of miR-34c inhibit malignant behavior such as invasion, migration, proliferation and epithelial-mesenchymal transition (EMT) in NPCs by targeting β-Catenin. In addition, we found alleviated radioresistance upon miR-34c overexpression or β-catenin knockdown in NPCs. Exosomes derived from miR-34c-transfected MSCs attenuated NPC invasion, migration, proliferation and EMT. Moreover, miR-34c-overexpressing exosomes drastically increased radiation-induced apoptosis in NPC cells.

**Conclusion:**

miR-34c is a tumor suppressor miR in NPC, which inhibits malignant behavior as well as radioresistance of tumor. Therefore, exogenous delivery of miR-34c to NPCs via MSC exosomes inhibits tumor progression and increases the efficiency of RT. Combination IR with miR-34c-overexpressing exosomes may be effective treatment for radioresistant NPCs.

## Background

Nasopharyngeal carcinoma (NPC) is a common malignant tumor in Southeast Asia, including southern area of China such as Guangdong and Guangxi province [1]. Many NPC patients already have advanced-stage NPC at diagnosis. Radiation therapy (RT) is the main treatment for NPC [2]. Some patients with early-stage NPC can be cured by RT, but due to malignant behavior and resistance to radiotherapy, 19% to 29% of patients still suffer local recurrence or distant metastasis after RT [2, 3]. Therefore, to explore pathogenesis of NPC, and thereby to develop current treatment for NPCs will definitely contribute to improved NPC prognosis.

Epithelial-mesenchymal transition (EMT) is an important process in tumor metastasis and recurrence [4, 5], and was recently found to promote tumor resistance to RT and chemotherapy [6]. EMT enables epithelial cells to acquire mesenchymal characteristics, including the deletion of epithelial morphology and markers (e.g., E-cadherin, desmoplakin, Muc-1, and cytokeratin-18) and the upregulation of mesenchymal markers (e.g., N-cadherin, vimentin, and fibronectin) [7]. Accordingly, our previous studies found that radioresistant NPC cells showed higher EMT level compared to normal NPC cells [8].

MicroRNAs (miRNAs) are small, noncoding RNAs that are altered in almost all types of cancer. microRNA-34c-5p (miR-34c) is a recognized tumor suppressor molecule [9]. It is reported that miR-34c inhibit EMT in several types of tumors such as colorectal [10], prostate [11], and lung cancer [12]. miR-34c has also been confirmed to enhance the sensitivity of cancer cells to radiation, thereby enhancing the effect of RT [13]. However, there are little relevant study on the role of the miR-34 family in NPCs.

Exosomes are secreted membrane-bound vesicles with a diameter of 30–150 nm that act as critical messengers for cell–cell communication [14]. Exosomes transfer proteins, nucleic acids and other molecules between cells. Some exosomes inhibit tumors and thus have the potential to treat tumor [15]. Mesenchymal stem cells (MSCs) are multipotent stromal cells that can differentiate into a variety of cell types, including osteoblasts, chondrocytes, myocytes and adipocytes, which are easily acquired from many tissues [16], They are reported to have the potential to treat a variety of diseases [17], including cancer [18]. Tumors are enriched in large numbers of MSC-derived exosome [19].

In the current study, we explored the role of miR-34c in NPC. miR-34c inhibited its downstream target, β-catenin, thereby reducing EMT in NPC. Moreover, miR-34c also suppressed the malignant behavior and alleviating radioresistance in NPC. Based on these data, we designed MSC-based therapy in which inhibited the radioresistance, invasion and metastasis of NPC.

## Materials and methods

### Patients and tissue samples

Samples were collected from 70 NPC patients who underwent radical RT at the Affiliated Tumor Hospital of Guangxi Medical University from January 2013 to December 2015 and analyzed for miR-34c expression (Table 1). In addition, clinical and pathological data (gender, age, Karnofsky Performance Status (KPS), tumor-node-metastasis (TNM) stage and pathologic type) were collected. All the patients were diagnosed by histopathology and had no prior RT or chemotherapy before tissue and tissue collection. All patients were treated by intensity modulation radiated therapy (IMRT) with a total dose of 68.2–72.32 Gy and a split dose of 2.18–2.26 Gy. Upon the completion of IMRT, the patients were divided into radiosensitive and radioresistance groups according to the observed therapeutic effects. Radioresistant NPC patients were defined as individuals with persistent disease (incomplete regression of primary tumor and/or neck lymph nodes) > 3 months after the completion of IMRT or with local recurrent disease at the nasopharynx and/or neck lymph nodes ≤ 12 months after the completion of IMRT. Radiosensitive NPC patients were defined as individuals without local residual lesions (complete regression) > 3 months after the completion of IMRT or local recurrent disease > 12 months after the completion of IMRT. The detailed clinical data are shown in Table 1. This study was conducted with the approval of the Ethics Committees of this hospital.

**Table 1:** Clinical and pathological parameters of the radiosensitivity and radioresistance groups

Classification	Radioresistance group (n = 41)	Radiosensitivity group (n = 29)	P*
Sex			
Male	35	23	0.752
Female	6	6	
Age			
<50	28	20	1.000
≥50	13	8	
KPS	90.00 ± 0.01	89.71 ± 1.69	0.321
Pathologic type			
Differentiated	8	5	1.000
Undifferentiated	33	24	
Clinical stage			
I	0	0	0.883
II	6	5	
III	14	11	
Iva + IVb	21	13	
T stage			
T1	2	2	0.693
T2	6	4	
T3	15	13	
T4	18	10	
N stage			
N0	1	0	0.694
N1	16	13	
N2	21	13	
N3	3	3	
Hemoglobin (g/l)	137.6 ± 15.5	139.8 ± 17.7	0.577
PGTVnx	71.99 ± 1.14	71.77 ± 1.18	0.437
PGTVnd	68.88 ± 2.15	68.20 ± 2.64	0.243
CTV1	60.74 ± 0.98	60.80 ± 0.99	0.809
CTV2	54.72 ± 0.87	54.77 ± 0.90	0.809

* There was no significant difference between the 2 groups

### Cell culture

NPC cell lines CME-2, 5–8F and 6–10B were maintained in our lab. CNE-2R cells (a radioresistant human NPC cell line) was constructed by fractionated radiation in vitro, as described in previous studies, and was maintained at the Cancer Laboratory of Guangxi Medical University. These cells were cultured in RPMI 1640 medium (GIBCO) with 10% FBS. The immortalized nasopharyngeal epithelial cell line NP-69 cells were cultured in keratinocyte serum-free medium (KSFM), supplemented with human recombinant epidermal growth factor (rEGF) and bovine pituitary extract. The immortalized nasopharyngeal epithelial cell line NP460 is established by a non-malignant nasopharyngeal biopsy tissue by telomerase immortalization technology, presented by Professor Cao Shihua of the University of Hong Kong, which is cultured in a mixture of KSFM medium (Invitrogen, Carlsbad, CA, USA) and EpiLife medium (Sigma, St Louis, MO, USA) pre-added EGDS in a ratio of 1:1. 293 T cells were cultured in Dulbecco’s modified Eagle’s medium, supplemented with 10% fetal bovine serum. All media were supplemented with 100 U/mL penicillin, 100 mg/mL streptomycin, and 2 mM GlutaMAX.

### Target prediction

The β-catenin gene ID: 1499, mature mRNA sequence (NM_001904) was obtained from the NCBI and queried by the miRNA target prediction sites TargetScan (Human) [20] (http://www.targetscan.org/) and miRbase (http://www.mirbase.org/) and miRDB [21] (http://mirdb.org/miRDB). The predicted binding strengths and scores were obtained. Only the miRNAs common to at least 2 searches were considered for further investigations.

### Immunohistochemistry (IHC)

Sections were obtained from paraffin-embedded tissues from sacrificed nude mice. The sections were heated, deparaffinized, rehydrated and placed in sodium citrate buffer (pH 6.0) for antigen retrieval, and the endogenous peroxidase activity was blocked with 3% hydrogen peroxide. The slides were blocked with 10% normal goat serum and incubated with primary antibody (Abcam) and Ki-67 (Cell Signaling Technology) at 4 °C overnight. The images were visualized by following standard protocols using a horseradish-peroxidase-conjugated secondary antibody and 3,3′-diaminobenzidine (DAB) as a substrate. Sections were incubated with normal rabbit serum to generate the negative controls. The slides were counterstained with haematoxylin, and typical images were obtained using a Leica DM 2500 microscope. The IHC-stained tissue sections were scored by three pathologists who were blinded to the clinical parameters, respectively. The percentage of immunostaining and the staining intensity (0, negative; 1+, weak; 2+, moderate; and 3+, strong) were recorded. An H-score was calculated using the following formula: [1 × (% cells 1+) + 2 × (% cells 2+) + 3 × (% cells 3+)] × 100. The maximum H-score would be 300, corresponding to 100% of cells with strong intensity.

### miR-34c overexpression and knockdown

Mature miR-34c mimics, inhibitors and the negative control miR were purchased from Genepharma (Shanghai, China). Lentiviruses encoding miR-34c, a miR-34c inhibitor and a scrambled control were purchased from Genechem (Shanghai, China). Transient cell transfections were performed using LipofectamineTM 3000 reagent (Thermo Fisher Scientific; USA) according to the manufacturer’ s protocol. Transfections with lentivirus were performed with an MOI = 30 in NPC cell lines and an MOI = 10 in the human MSCs.

### Exosome isolation and identification

Exosomes from MSCs were isolated from conditioned medium from MSCs, and the procedures used for isolation were performed as previously described [31]. The exosomes were stored at − 80 °C and verified by electron microscopy and nanoparticle tracking technology (NanosightTM).

### Transwell invasion and migration assays

NPC cells were added to the top chamber in serum-free media. For the invasion assay, 1 × 10^5^ cells were seeded into 24-well plates with Matrigel-coated Transwell inserts (Corning). For the migration assay, the cells were seeded onto membranes in the absence of Matrigel. The bottom chamber was filled with 1640 containing 10% FBS. After 13 h or 24 h of incubation, the cells in the top chamber were removed using a cotton swab, and the membrane was fixed in 4% paraformaldehyde for 15 min and stained with crystal violet for 15 min. Five fields of adherent cells from each well were photographed randomly.

### Luciferase reporter assay

The reporter genes containing pGL3-β-catenin and pGL3-mutβ-catenin were synthesized by Bio-Asia (Jinan, China). The NPC cells were co-transfected with the luciferase reporters and the miR-34c mimics, and 48 h later, the activity of the reporter protein was measured using a luciferase assay kit (Promega; USA) according to the manufacturer’s instructions.

### Western blotting

The harvested cells were lysed using heat denaturation in RIPA cell lysis buffer. The protein lysates were loaded and separated using SDS-PAGE and then transferred to a polyvinylidene difluoride (PVDF) membrane. The blots were incubated with primary antibodies against β-catenin (1:500 dilution, Proteintech; China), N-cadherin (1:1000 dilution, Abcam, USA), E-cadherin (1:1000 dilution, Abcam, USA), MMP9 (1:1000 dilution, Abcam, USA), ROCK1 (1:1000 dilution, Abcam, USA), Bcl-2 (1:1000 dilution, Abcam, USA), bax (1:1000 dilution, Abcam, USA) and GAPDH (1:1000; Cell Signaling Technology; USA). To visualize the protein bands, enhanced chemiluminescence (ECL, Millipore, Bedford, USA) was used. The intensity of the protein bands was normalized to GAPDH and analysed using ImageJ software.

### Quantitative real-time PCR (qRT-PCR)

Total RNA was isolated from NPC cells using Trizol reagent (Invitrogen, Life Technologies). Reverse transcription was performed using 2 μg of total RNA and the High Capacity cDNA Reverse Transcription Kit (Applied Biosystems) according to the manufacturer’ s protocol. The cDNA was subject to real-time PCR using the Mx-3000P Quantitative PCR System (Stratagene). The primers for miR-34c were 5′-ACACTCCAGCTGGGAGGCAGTGTAGTTAGCT-3′ and 5′-CTAACTGGTGTCGTGGAGTCGGCAATTCAGTTGAGGCAATCAG-3′. The primers for U6 were 5′-CAGCACATATACTAAAATTGGAACG-3′ and 5′-ACGAATTTGCGTGTCATCC-3′. The primers for β-catenin were 5′-CCCACTAATGTCCAGCGTTT-3′ and 5′-AATCCACTGGTGAACCAAGC-3′. The primers for GAPDH were 5-GCACCGTCAAGGCTGAGAAC-3 and 5-TGGTGAAGACGCCAGTGGA-3. The relative mRNA expression was normalized to that of GAPDH, while the relative miR expression normalized to that of U6. All reactions were performed in a 20 μL volume in triplicate. Data were assessed using the 2-ΔΔCt method. PCR amplification was performed as follows: 15 s at 95 °C, 15 s at 51–58 °C (depending on the gene) and 45 s at 72 °C for 40 cycles.

### CCK-8 assay

The Cell Counting Kit-8 (CCK-8) assay was used to assess cell viability. Cells were seeded in 96-well plates at 3 × 103 cells/well and allowed to attach overnight. After treatment, the cells were incubated with 10 µg/mL CCK-8 solution (Dojindo, Japan) for 1 h in a humidified chamber containing 5% CO^2^ at 37 °C. Absorbances at 450 nm were read on a microplate reader (Bio-Rad). Five replicate wells were used to assess each group, and three independent experiments were conducted.

### Colony formation assay

A cellular suspension was seeded at 600 cells/well into a 6-well plate that was then incubated at 37 °C in 5% CO2 for 14 days until visible clones appeared. Then, the cell culture medium was discarded, and the cells were washed with phosphate-buffered saline (PBS) three times. The cells were fixed with 4% paraformaldehyde (Solarbio) for 15 min and then stained with 0.1% crystal violet (Sigma) for 15 min before washing with deionized water and air-drying. Colonies were counted under a light microscope.

Colony formation assays were conducted to evaluate the radiosensitivity of the cells after IR. Suspensions containing 200, 200, 400, 800, 1000, and 2000 cells were seeded into five of the six-well plates and individually exposed to doses of 0, 2, 4, 6, 8 and 10 Gy, respectively, with a 6 MV X-ray beam from an Elekta linear accelerator (Precise 1120; Elekta Instrument AB, Stockholm, Sweden) at a dose rate of 220 cGy/min. Then, the cells were incubated for another 14 days until colonies appeared. The colonies were then fixed with carbinol for 15 min and stained with 0.1% Giemsa (AppliChem, Germany) for 30 min. Colonies containing more than 50 cells were counted. All experiments were conducted three times. Dose–response curves were analyzed using the linear quadratic (LQ) model with GraphPad Prism 7.0 software with the following formula: S = exp^−(αd+βd^2)^, where S represents the number of cell survival, d represents a single dose, α represents a linear effect, and β represents a square effect.

### Apoptosis assessment

Cell apoptosis was assessed by using Annexin V APC/7-AAD apoptosis kit (BD Pharmingen, USA). Samples were trypsinized, washed with ice-cold PBS, and stained with 5 μL Annexin V APC and 5 μL 7-AAD according to the manufacturer’s instructions. Analysis was carried out immediately on a FC500 flow cytometry system and FlowJo software. All samples were assayed in triplicate. The predicted results were that regular cells (APC−/7-AAD−), necrotic cells (APC+/7-AAD+), and apoptotic cells (APC+/7-AAD−).

### Xenograft mouse model

BALB/c nude mice (4–5 weeks old) were from SLAC Laboratory Animals (Shanghai). After disinfection by 75% alcohol of right hind-limb groin skin, 0.2 mL of cell suspension (1 × 107 cells/mL) were subcutaneously injected into the right groin. Tumor formation was determined, and the long (a) and short (b) diameters of each tumor were measured with a Vernier caliper every 3 days after tumor formation. Tumor volumes were calculated as V = 0.5 × a × b^2^ and used to generate tumor growth curves. When the transplanted tumor reached about 0.8–1 cm in length, the nude mouse was fixed on a treatment bed, with the right hind limb stretched to expose the tumor to the light field. Tumor irradiation at 8 Gy was performed with a 6-MV X-ray beam from an Elekta linear accelerator (Stockholm, Sweden). The ray was applied with an SSD of 100 cm and a dose rate of 94.45 cGy/min. The irradiation field targeted the mouse hind limb bed site, with lead protection used for the remaining animal parts. The transplanted tumors were irradiated, with a continuous observation. Tumor growth rate (R) were calculated as R = (V_t_ − V_0_)/V_0_ (V_t_ is the tumer volume on the day, V_0_ is the tumer volume 1 day before irradiation).

### Statistical analysis

Statistical analyses were performed using GraphPad Prism 7.0 software (GraphPad, USA) and SPSS 20.0 software (IBM, USA). All data are presented as the mean ± standard error of three independent experiments and were evaluated by two-tailed unpaired Student’s *t* test. Categorical data are reported using frequencies and percentages and were evaluated using Pearson’ s Chi square test or Kruskal–Wallis analysis. For in vitro and in vivo experiments, a t-test or analysis of variance was used to evaluate the differences between different groups. All statistical tests were two-sided, and significance was assigned at P < 0.05.

## Results

### miR-34c is downregulated in NPC tissues and cell lines and negatively associated with radioresistance

**Fig. 1:**
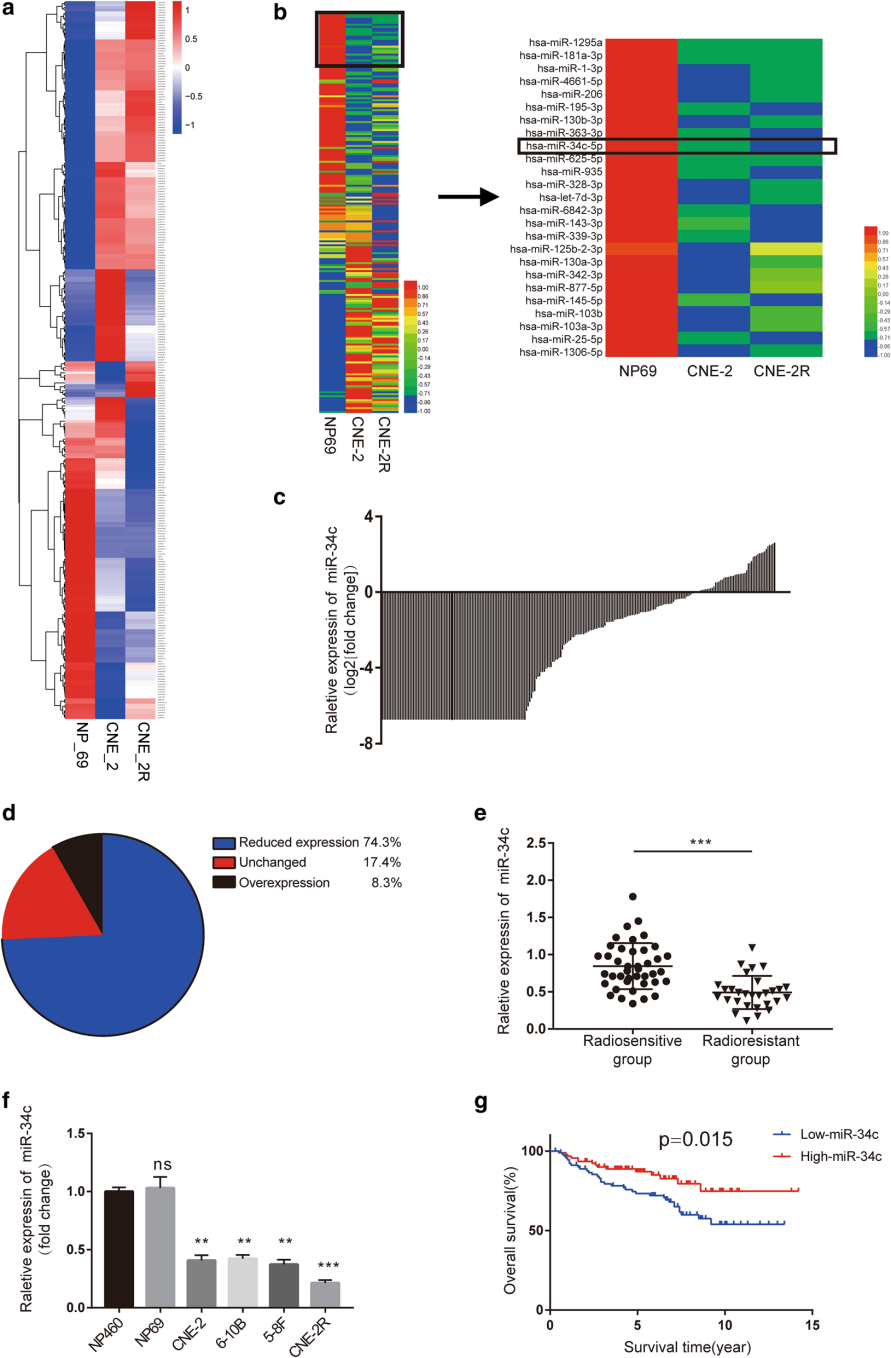
Expression of miR-34c is downregulated in NPC cells and tissues and associated with radioresistance in patients with NPC. **a**, **b** Heatmap displaying miRNA expression in NP69, CNE-2, and CNE-2R cells; miR-34c was selected for further study. **c** Waterfall plot showing the relative expression of miR-34c in NPC tissues (NCBI/GEO/GSE70970). **d** Pie chart displaying the miR-34c levels in NPC tissues; a fold change > 2 or < 1/2 in relative miR-34c expression was defined as significant. **e** The miR-34c levels in tissues from radioresistant patients (n = 29) and radiosensitive patients (n = 41) were measured by qRT-PCR. **f** The miR-34c levels in NP-460, NP69, CNE-2, 6–10B, 5–8F and CNE-2R cells were measured by qRT-PCR. **g** Kaplan–Meier curve for overall survival in the cohort of 185 NPC patients (NCBI/GEO/GSE70970). Patients were divided into two groups based on miR-34c level in each sample, and the median ratio value was chosen as the cutoff point

To find the key inhibiting miRNAs for NPC initiation, progression and radioresistance, we conducted miRNA sequencing for NP69, CNE-2 and CNE-2R cells (Fig. 1a). We chose the top 200 most expressed miRNAs in NP69 cells (Fig. 1b, left panel), and listed the drastically decreased 25 miRNAs in CNE-2 cells compared to NP69 cells (Fig. 1b, right panel). To verify these sequencing results in clinical samples, miRNA expression in a Gene Expression Omnibus (GEO) database (NCBI/GEO/GSE70970) or in 70 NPC samples from Guangxi Medical University Cancer Hospital (Table 1) were analyzed. We found that miR-34c significantly decreased in NPC tissue samples compared to normal nasopharynx samples (Fig. 1c, d), and expressed even lower in radioresistance NPC tissue samples compared to their radiosensitive counterparts (Fig. 1e), which in accordance with the miRNA sequencing results for cell lines (Fig. 1b). Moreover, the results for qRT-PCR confirmed that the normal nasopharynx cell lines displayed the highest miR-34c expression whereas the radioresistance NPC cell line displayed the lowest (Fig. 1f). Survival analysis for clinical data in GEO database indicate that higher expression of miR-34c significantly shorten the survival time for NPC patients (Fig. 1g).

In summary, these results suggest that lower expression of miR-34c is related to development and radioresistance for NPC.

### Overexpression of miR-34c inhibits NPC EMT and cell proliferation

Since miR-34c is decreased in NPC, we asked whether miR-34c acts as a tumor suppressor in NPC. We subsequently explored the function of miR-34c in vitro by gain-of-function assays.

**Fig. 2:**
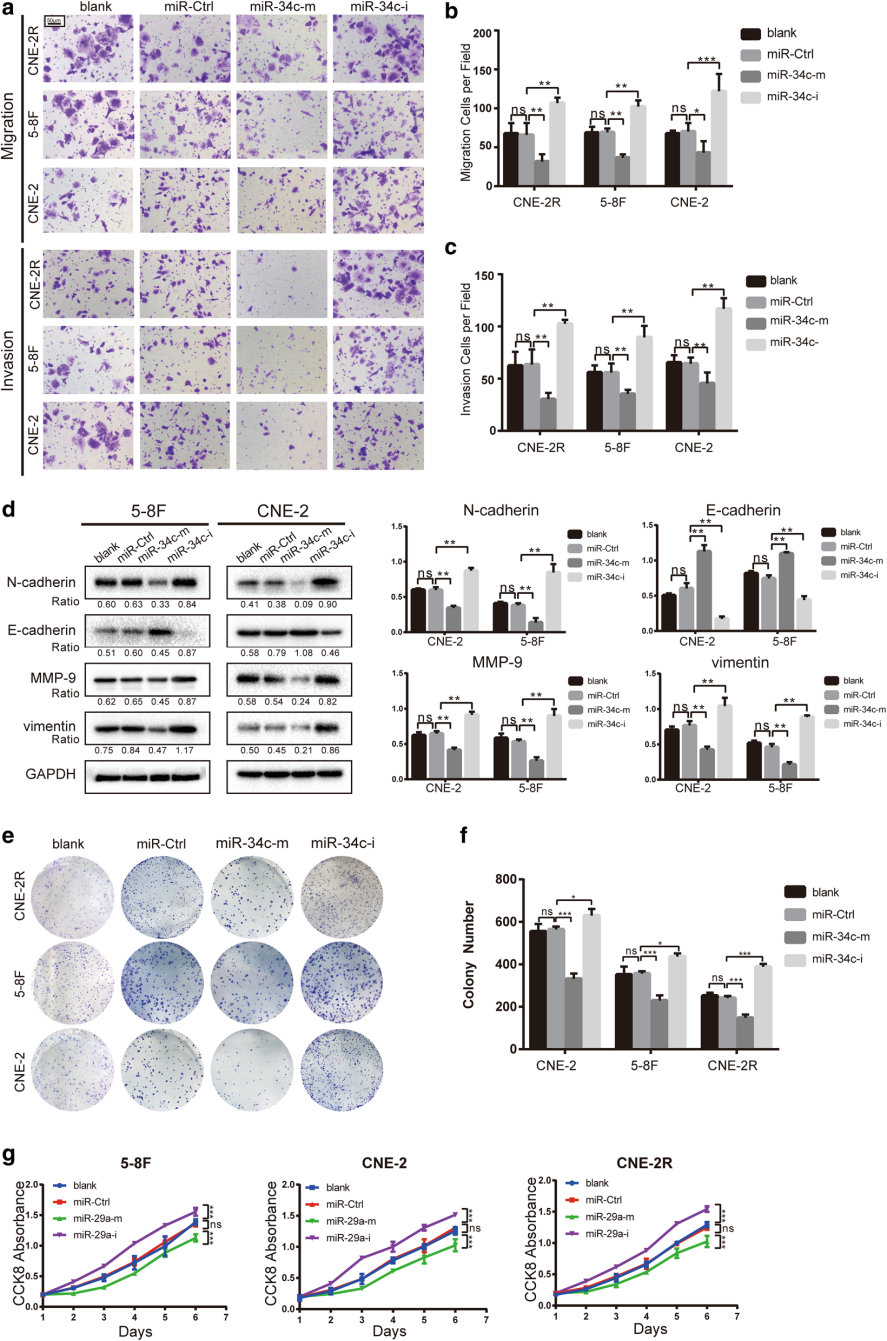
Overexpression of miR-34c inhibits migration, invasion and proliferation and limits EMT in NPC cells. CNE-2, 5–8F and CNE-2R cells were transfected with miR-34c control (miR-Ctrl), mimics (miR-34c-m) or inhibitors (miR-34c-i). **a** Cell migration was assessed using Transwell migration and invasion assays (10 times magnification). **b, c** The number of cells per field in a migration and invasion assay. **d** Western blot analysis of EMT-related markers in CNE-2 and 5–8F cells transfected with lentivirus from each group. Gray value shows in the right panel. **e** Photos of colonies of NPC cells in colony formation assays. **f** The number of colonies in different groups. **g** Cell proliferation in each group of CNE-2, 5–8F and CNE-2R cells was measured by CCK8 assay (the results were reproduced in three independent experiments.*P < 0.05; **P < 0.01; ***P < 0.001; ns: no statistical significance)

The impact of miR-34c on cell migration and invasion was detected using Transwell assays. miR-34c overexpression significantly diminished cell migration and invasion, whereas after inhibition of miR-34c, all NPC cell lines displayed enhanced invasion and migration abilities (Fig. 2a–c).

We then detected the key factors related to migration and invasion. We found a downregulation of mesenchymal markers N-cadherin, Vimentin and MMP9, and a simultaneously upregulation of epithelial marker E-cadherin after miR-34c overexpression, which indicate an inhibition of EMT in NPC cells (Fig. 2d). As EMT is a process driving invasion and metastasis in carcinoma [25], we herein confirm that miR-34c ameliorate migration and invasion in NPC cells by inhibiting EMT process.

Next, colony formation assay and CCK-8 assay were used to assess the effect of miR-34c on cell proliferation. The results show that miR-34c markedly impeded proliferation of NPC cells, whereas inhibition of miR-34c significantly promoted the viability (Fig. 2e–g).

In summary, these results show that miR-34c inhibit EMT and proliferation in NPC cells.

### miR-34c induces apoptosis and alters the radiosensitivity of NPC cells

Since our results have revealed that lower expression of miR-34c is associated with radioresistance in NPC cells and clinical samples (Fig. 1a, d), we conducted further experiments to determine the role of miR-34c on radiosensitivity of NPC cell lines.

**Fig. 3:**
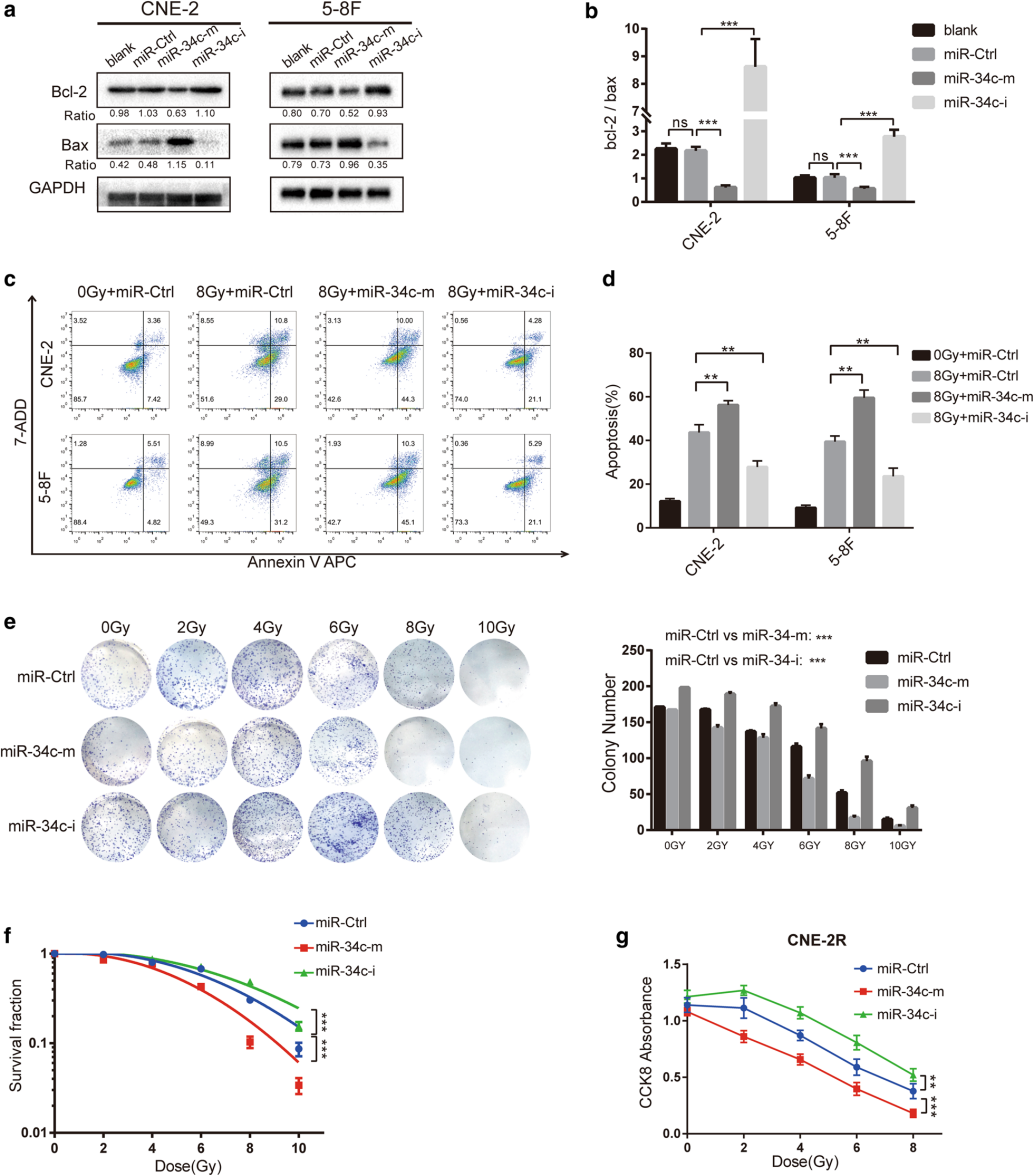
Overexpression of miR-34c promotes apoptosis and limits the radioresistance of NPC cells. **a** Western blot analysis of the apoptosis markers Bcl-2 and bax in CNE-2 and 5–8F cells. **b** Bar graph showing the ratio of Bcl-2/bax in each group. **c** The effect of miR-34c on cell apoptosis was analyzed by flow cytometry with APC/7-AAD staining. **d** The proportion of apoptotic cells. **e** Colony formation assays assessing the radiosensitivity of each group and the qualified number of colonies in each group. **f** Curves showing radio resistance were generated and fit with an LQ model. **g** The effects of miR-34c on the survival of CNE-2R after radiation was determined by CCK-8 assay (the results were reproduced in three independent experiments. *P < 0.05; **P < 0.01; ***P < 0.001; ns: no statistical significance)

Firstly, we explore the role of miR-34c on apoptosis. Expression of bcl-2 and bax in NPC cells were determined by Western blotting (Fig. 3a, b). We found a decrease of bcl-2/bax ratio in the miR-34c mimics group and an increased bcl-2/bax ratio after miR-34c inhibitor transfection. Since the bcl-2/bax ratio is negatively corelated to the level of apoptosis, these results suggest that miR-34c could induce apoptosis in NPC cells.

We then performed flow cytometry with APC/7-AAD staining to detect the apoptosis induced by irradiation. 8 Gy irradiation significantly induce apoptosis in NPC cells (Fig. 3c, d). We found that miR-34c overexpression could increase the irradiation-induced apoptosis, and on the contrary, the inhibition of miR-34c offset the apoptosis induced by radiotherapy in NPC cells (Fig. 3c, d). These results indicate that under the condition of irradiation, miR-34c still have the capacity to induce apoptosis in NPC cells.

To determine whether miR-34c could reduce radioresistance of NPC cells, we then conducted colony formation assay using CNE-2R cell line. Overexpression of miR-34c significantly decrease the colony numbers after irradiation treatments (Fig. 3e). The cell survival curves were fitted with the L-Q model and used to illustrate the sensitivity to radiotherapy. We found a significantly reduced survival fraction of NPC cells upon miR-34c overexpression where the SF value reduced from 0.09 ± 0.01 to 0.03 ± 0.005, while the SF value of miR-34c inhibiting group is 0.16 ± 0.015 (P < 0.001) indicating that miR-34c suppresses resistance to radiotherapy in NPC cells (Fig. 3f). In addition, the results for CCK-8 assay suggest that miR-34c overexpression markedly reduced the proliferation of NPC cells treated by different doses of radiation (Fig. 3g).

To sum up, these results demonstrate that miR-34c promote apoptosis and inhibit resistance to radiotherapy in NPC cells.

### miR-34c targets β-catenin in NPC

**Fig. 4:**
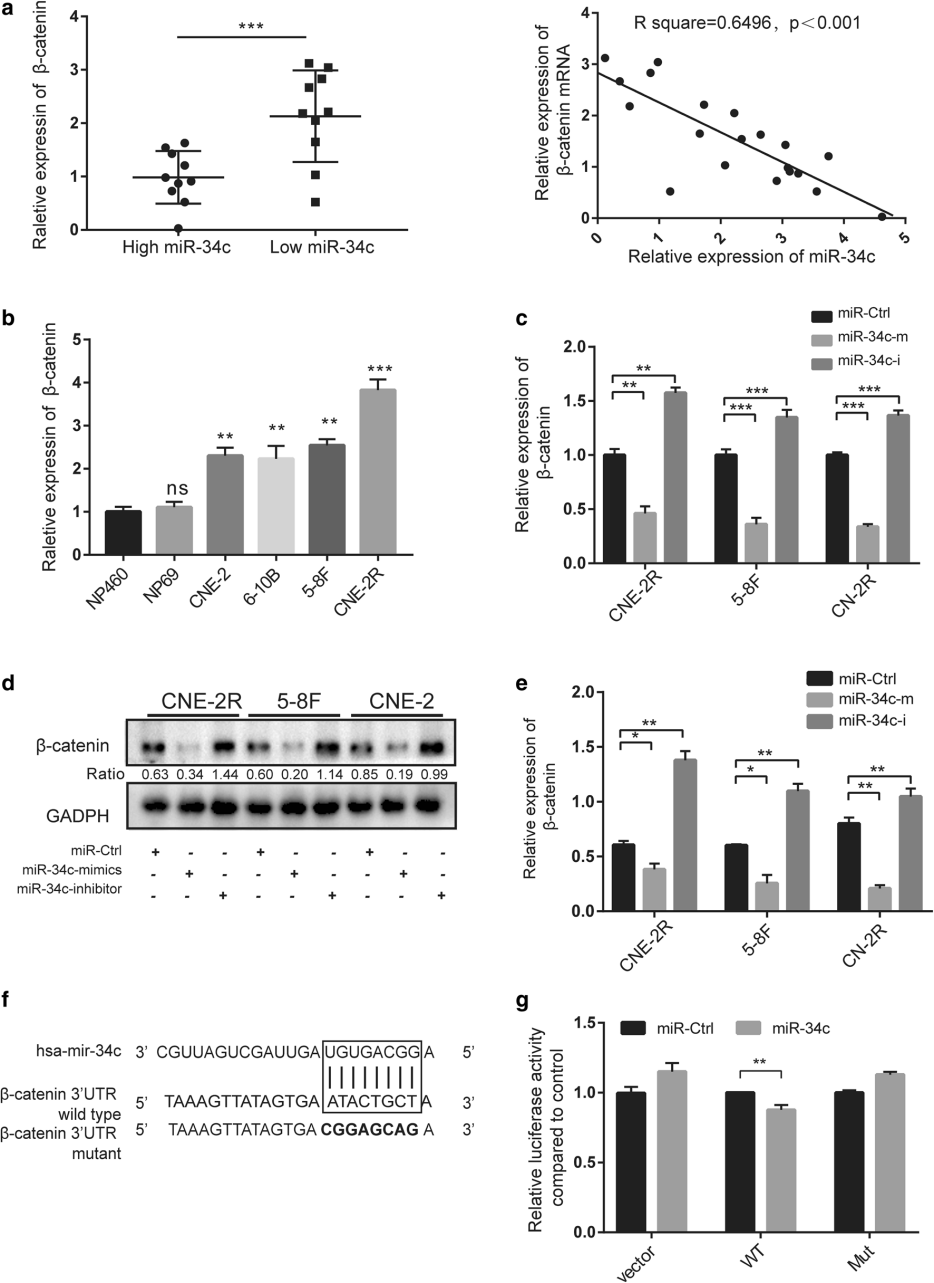
β-Catenin is a direct target of miR-34c. **a** Correlation between β-catenin mRNA and miR-34c levels in the serum of 20 NPC patients measured by qRT-PCR; expression was normalized to the mean level in three normal serum samples. **b** Expression of β-catenin in different NPC cells determined by qRT-PCR. **c** β-catenin was measured by qRT-PCR in miR-34c-transfected NPC cells. **d** After 48 h of transfecting miR-34c, β-catenin protein expression was detected by Western blot analysis. **e** β-Catenin expression level determined by qRT-PCR 48 h after transfection with miR-34c. **f** Graphical representation indicating miR-34c interaction sites within the 3′-UTR of β-catenin mRNA with boxes. Mutations were generated in the β-catenin 3′-UTR site complementary to the seed sequences of miR-34c. **g** The β-catenin 3′-UTR with a putative wild-type (WT) miR-34c-binding site or a binding site mutated at the 3′-UTR region (Mut) were cloned downstream of the CMV promoter in the pmiR-REPORT vector. Luciferase activity was measured after cotransfection of the reporter constructs with WT- or Mut-interacting sites or miR-Ctrl in HEK293T cells (data are presented as the mean ± SD of three independent experiments. *P < 0.05; **P < 0.01; ***P < 0.001; *ns* no statistical significance)

To determine the target of miR-34c in NPC, we used two miRNA target prediction tools. Both TargetScan (http://www.targetscan.org/) and miRbase (http://www.mirbase.org/) predict β-catenin (CTNNB1) to be a potential target of miR-34c. Closely related to EMT in many tumors, β-catenin is a key molecule in the Wnt/β-catenin pathway [22]. We examined the expression of β-catenin and miR-34c in tissue of 20 NPC patients by qRT-PCR. As shown in Figure A (left panel), tissue with high β-catenin level had lower expression of miR-34c. Correlation analysis was performed, and the straight fitted line indicated a negative correlation between the expression levels of β-catenin and miR-34c (Fig. 4a right panel). Moreover, we found that NPC cell lines displayed higher levels of β-catenin mRNA in comparison with normal nasopharynx cells (Fig. 4b). We then detected both the mRNA and protein level of β-catenin after transfection of miR-34c mimics and inhibitors. Expression level of β-catenin decreased significantly after miR-34c overexpression, whereas4 inhibition of miR-34c elevated the β-catenin expression (Fig. 4c–e).

To confirm whether β-catenin is a direct target of miR-34c, we performed luciferase reporter assays in cells transfected with the 3′-UTR of β-catenin mRNA containing miR-34c target sequences (Fig. 4f). The luciferase activity was downregulated by miR-34c significantly in wild type (WT) group but not in the mutation (Mut) group, indicating that miR-34c directly targeted β-catenin mRNA (Fig. 4g).

### miR-34c inhibits NPC growth and radioresistance in vivo

**Fig. 5:**
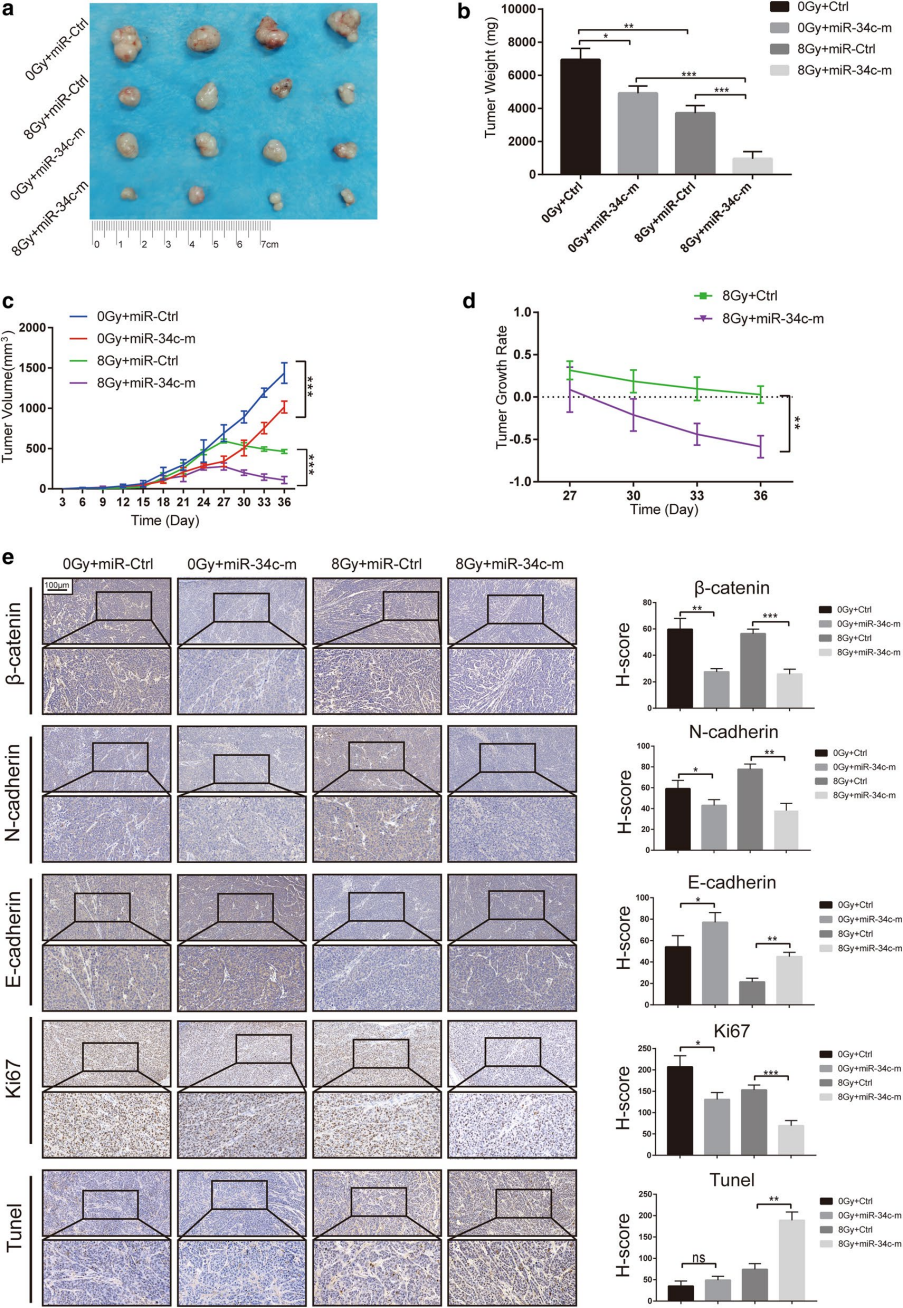
Effect of miR-34c overexpression on migration, invasion, proliferation and radioresistance in vivo. **a** CNE-2R cells stably overexpressing miR-34c or control were injected subcutaneously in nude mice. The mice were treated with RT or not at day 24 (n = 4 in each group). **b**, **c** The tumor volumes were monitored every 3 days, and after sacrifice, tumor weights were measured. **d** Graph of tumor growth rate. **e** Left panel: Representative images of β-catenin, Ki67, E-cadherin, and vimentin immunohistochemistry (IHC) staining at 20-fold and 40-fold magnification a nd TUNEL assay of tumor samples from each group. Right panel: H-score of each group

To explore the role of miR-34c on NPC growth and radioresistance in vivo, we transplanted the radioresistant CNE-2R cells subcutaneously into nude mice and irradiation was performed 24 days after transplantation (Fig. 5a). We found that as expected, miR-34c alone could mildly reduce the tumor volume and weight. However, in the context of irradiation, miR-34c overexpressing tumors displayed drastically decreased tumor volume and weight, indicating that the inhibiting ability of miR-34c on tumor growth were amplified when irradiation was used (Fig. 5b, c). This result somehow suggested that after irradiation, the significant decrease of tumor volume and weight in miR-34c overexpression group was a consequence of both the decreased cell growth and the reduced radioresistance. To further confirm the inhibition of radioresistance, we studied the tumor growth rate based on the tumor volume curve in Fig. 5c, d. We confirmed that NPC overexpressing miR-34c displayed drastically higher sensitivity to radiotherapy (Fig. 5d). In accord with the results above, the IHC staining for Ki67 indicated that miR-34c overexpression decreased the proliferation of tumor in vivo, and these effects became more obvious in the context of irradiation (Fig. 5e). Interestingly, we found that Tunel positive cells were rather few in the 0 Gy groups, even in tumor overexpressing miR-34c. The 8 Gy irradiation elevated the Tunel positive cells and, most notably, miR-34c significantly amplified this effect (Fig. 5e). These results suggested that miR-34c could impact radioresistance by facilitating the irradiation-induced apoptosis in vivo. Furthermore, miR-34c decreased the expression of β-catenin, which confirmed the results in Fig. 4 in vivo (Fig. 5e). The decrease of N-cadherin and increase of E-cadherin suggested an inhibition of EMT upon miR-34c transfection (Fig. 5e).

Altogether, these results demonstrate that miR-34c could inhibit radioresistance and EMT in vivo.

### β-Catenin mediates miR-34c-induced inhibition of EMT, proliferation and radioresistance

**Fig. 6:**
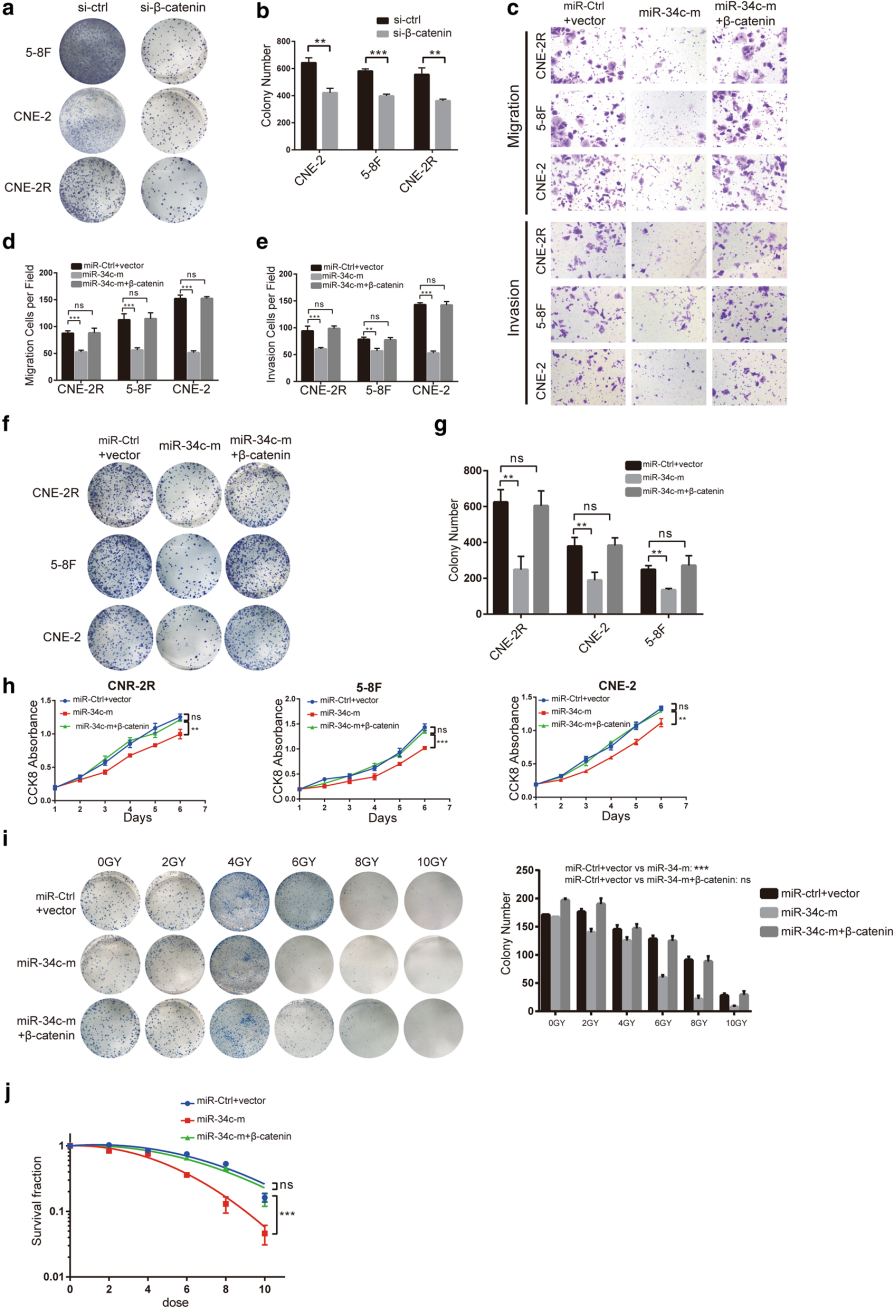
β-catenin mediates miR-34c-induced inhibition of EMT, proliferation and radioresistance in vitro. **a**, **b** Colony assays showing the effect of β-catenin on NPCs proliferation. **c** Cell movement ability was determined by Transwell assays (10 times magnification). **d**, **e** The number of cells per fields in a migration and invasion assay. **f** Photos of colonies of CNE-2, 5–8F and 6–10B cells in colony formation assays. **g** Bar graph showing the number of colonies in different groups. **h** Cell viability in each group of NPC cells was analyzed by CCK8 assay. **i** Colony formation assays assessing the radiosensitivity the qualified number of colonies of each group. **j** Curves showing the radiosensitivity of each group were created and fit with an LQ model. The results are presented as the mean ± SD (n = 3) (*P < 0.05; **P < 0.01; ***P < 0.001; ns: no statistical significance)

We transfected CNR-2, 5–8F, and CNR-2R cells with si-β-catenin and found that knockdown of β-catenin could inhibit the proliferation of NPC (Fig. 6a, b). Additionally, rescue experiments were conducted to explore the relationship between miR-34c and β-catenin.

We found that after overexpression of β-catenin in miR-34c-transfected NPC cells, the inhibitory effects of miR-34c on migration and invasion was offset (Fig. 6c–e). The proliferation and colony formation inhibited by miR-34c were also restored after β-catenin overexpression (Fig. 6f–h). To further detect the role of β-catenin on radioresistance in radioresistant CNE-2R cells, we performed colony formation assay and CCK8 assay. We found that although the SF value reduced from 0.16 ± 0.025 to 0.05 ± 0.015 after treatment of miR-34c (P < 0.001) indicating that miR-34c inhibited the radioresistance in NPC cells, transfecting of β-catenin significantly reversed the inhibitory role of miR-34c turning the SF value back to 0.15 ± 0.035 (Fig. 6i, j).

These results suggest that the anti-tumor role of miR-34c in NPC cells was mediated by β-catenin, that β-catenin overexpression could counteract the function of miR-34c to inhibit NPC migration, invasion, proliferation and radiation resistance.

### Transfected MSCs transfer miR-34c via exosomes and inhibit the development of NPC

Since we demonstrated the antitumor role of miR-34c in NPC, we further investigated whether artificially increasing the expression of miR-34c has a therapeutic effect in NPC.

**Fig. 7:**
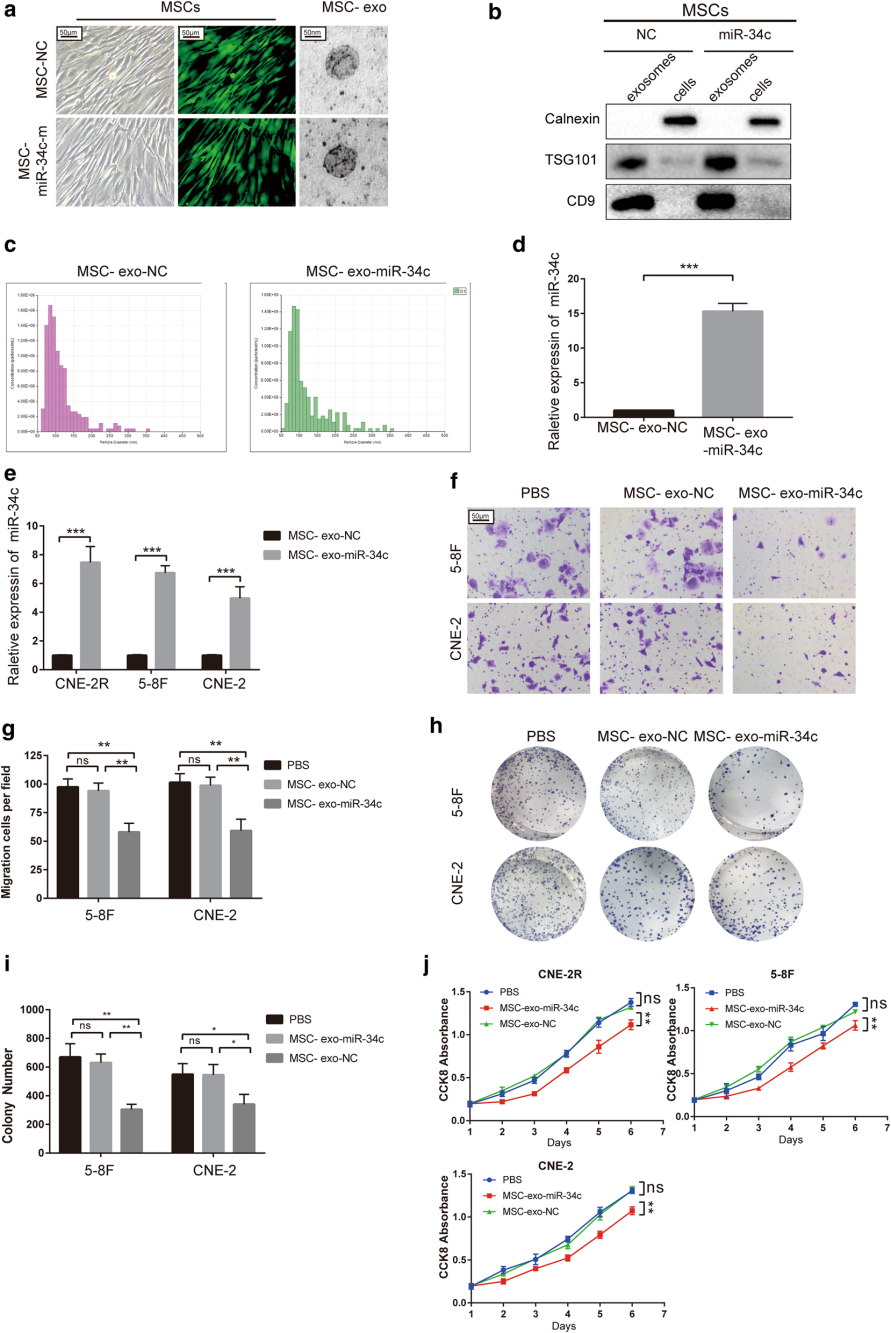
Exosomes derived from MSCs overexpressing miR-34c attenuate malignant behavior in NPC. **a** Representative images of human MSCs transfected with miR-34c or NC nucleotide sequences and corresponding electron microscopic images of exosomes. **b** Western blot analysis showing the presence of TSG101 and CD9 and the absence of calnexin in MSC-derived exosomes. **c** Particle size distribution of exosomes measured by Nanosight. **d** PCR analysis of miR-34c levels in exosomes. **e** miR-34c expression in NPCs detected by qRT-PCR after treatment with PBS or miR-34c-overexpressing (MSC-exo-miR-34c) or control (MSC-exo-NC) exosomes. **f** Migration ability detected by Transwell assay. **g** The number of cells per field in migration and invasion assays. **h** Photos of colonies of each group in colony formation assays and **i** a bar graph showing the number of colonies in different groups. **j** Cell proliferation in each group was measured by CCK8 assay (the results were reproduced in three independent experiments.*P < 0.05; **P < 0.01; ***P < 0.001; ns: no statistical significance)

First, we stably transfected MSCs with a lentivirus vector expressing miR-34c and the transfection efficiency was verified (Fig. 7a). The GFP positive cells accounted for 80–85% in each group (Additional file 1: Figure S1). We then used an ultrafiltration method to separate the exosomes, which were then photographed using an electron microscope (Fig. 7a) and found to have a diameter of approximately 100 nm. As shown by Western blotting, the exosome markers TSG101 and CD9 were highly expressed, while the endoplasmic membrane marker calnexin was not expressed (Fig. 7b). Nanoparticle tracking technology (Nanosight™) showed sharp peaks at 50–100 nm, demonstrating extracted nanovesicles were exosomes (Fig. 7c). The miR-34c expression level in the exosomes was measured by qRT-PCR (Fig. 7d), which indicated that exosomes from MSCs overexpressing miR-34c contained higher level of miR-34c accordingly. We then detected the changing of miR-34c expression after MSC exosomes treatments in NPC cells (Fig. 7e). The expression level of miR-34c in NPC cells upregulated significantly 48 h after treatment of exosomes derived from miR-34c transfected MSCs (MSC-exo-miR-34c) (Fig. 7e).

**Fig. 8:**
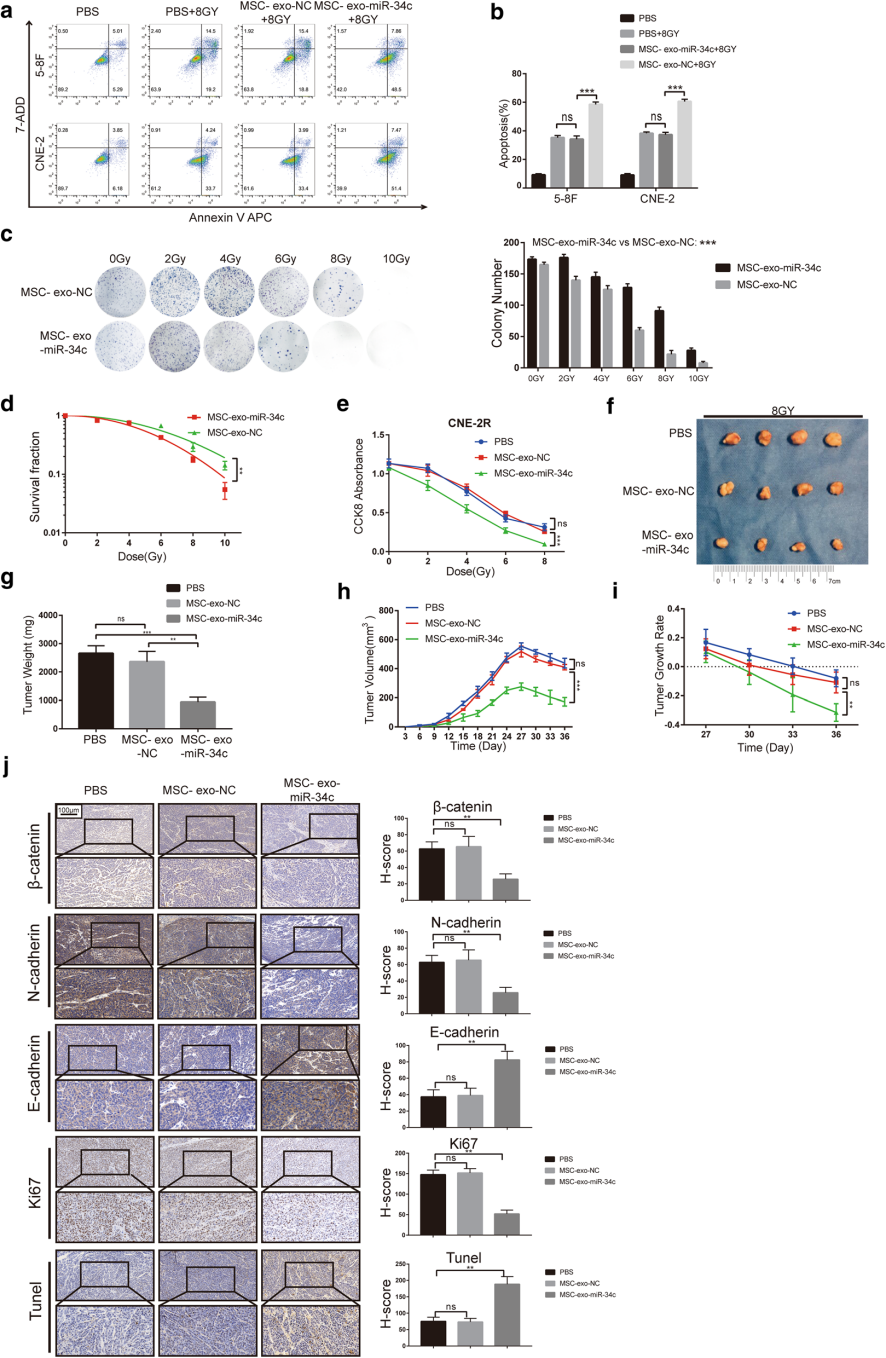
Exosomes derived from MSCs overexpressing miR-34c attenuate radioresistance of NPC in vivo and in vitro. **a** Cell apoptosis in each group was analyzed by flow cytometry with APC/7-AAD staining. **b** Bar graph showing the rate of apoptosis in cells. **c** Colony formation assays assessing the radiosensitivity and the qualified number of colonies in each group. **d** Curves showing radioresisitance were generated and fit with an LQ model. **e** CCK-8 assay assessing the survival of CNE-2R after radiation. **f** CNE-2R cells were treated with PBS, and exosomes were injected subcutaneously into nude mice (n = 4 in each group). The mice were treated with RT at day 24. **h** Tumor volumes were monitored every 3 days, and **g** after sacrifice, tumor weights were measured. **i** Tumor growth rate was calculated. **j** Left panel: Representative images showing the IHC staining of tumor samples from different groups for β-catenin, N-cadherin, E-cadherin, Ki67, and TUNEL assays at 20-fold and 40-fold magnification. Right panel: H-score of each group (*P < 0.05; **P < 0.01; ***P < 0.001; *ns* no statistical significance)

Next, we pretreated NPC cells with exosomes derived from miR-34c-transfected MSC (MSC-exo-miR-34c) and empty vector group (MSC-exo-NC) for 48 h. A Transwell assay indicated that MSC-exo-miR-34c significantly reduced migration in NPC cells (Fig. 7f, g). Results of colony formation experiments and CCK8 assay demonstrated that MSC-exo-miR-34c significantly reduced the proliferation of NPC cells (Fig. 7h–j). Furthermore, the treatment by MSC-exo-miR-34c increased the apoptosis in NPC cells upon 8 Gy radiotherapy (Fig. 8a, b) and decreased the surviving colony after different dosages of irradiation (Fig. 8c). The sensitivity to radiotherapy were detected by cell survival curves (Fig. 8d), the SF value reduced from 0.14 ± 0.023 to 0.05 ± 0.015 after treatment of miR-34c exosome (p < 0.001). We found that after treatment of MSC-exo-miR-34c, NPC cells displayed decreased level of radioresistance, whereas treatment of MSC-exo-NC seemed not to have the similar effects (Fig. 8d). In addition, the results for CCK-8 assay suggest that MSC-exo-miR-34c markedly reduced the proliferation of NPC cells treated by different dosages of radiation (Fig. 8e).

We then performed the in vivo study to detect the role of MSC-exo-miR-34c on NPC radioresistance (Fig. 8f). CNE-2R cells were transplanted subcutaneously and tumor bearing mice acquired PBS, MSC-exo-NC or MSC-exo-miR-34c injections for 14 consecutive days since day 7 after transplantation. All groups were performed radiotherapy with 8 Gy dose. After the miR-34c exosome treatment, the tumors are more hollowed indicating the increased apoptosis (Additional file 1: Figure S3A). We found that miR-34c exosome alone could mildly reduce the tumor volume and weight (Additional file 1: Figure S2). However, in the context of irradiation, the tumor volume and weight reduced drastically, indicating that the inhibiting ability of miR-34c exosome on tumor growth were amplified combined with irradiation (Fig. 8g, h). And the graph for tumor growth rate upon radiotherapy indicated that this growth inhibition was, at least partly, due to the decreased resistance to radiotherapy in NPC treated by MSC-exo-miR-34c (Fig. 8i). IHC staining indicated an increase of Tunel positive cells but a decrease of Ki67 positive cells in the MSC-exo-miR-34c treated group (Fig. 8j). N-cadherin and β-catenin were downregulated and E-cadherin was elevated, suggesting that MSC-exo-miR-34c may also inhibit EMT by targeting β-catenin in vivo (Fig. 8j). HE staining was performed to detect tumor budding which can be considered as a symbol of metastasis and a marker of EMT (Additional file 1: Figure S3B) indicating the miR-34c exosome inhibiting effect on EMT as well as metastasis.

These results demonstrated that MSC-exo-miR-34c, but not the MSC-exo-NC, have the ability to inhibit NPC cell migration, invasion, proliferation and radioresistance in vitro and in vivo.

## Discussion

miR-34c is an important tumor suppressor in various tumors [23, 24]. There is also evidence that miR-34c may enhance the radiation sensitivity of cancer cells, thereby enhancing the effect of RT. In the NPC field, only one article indicates that miR-34c can inhibit the growth and metastasis of NPC [23], but the specific relationship between miR-34c and radiation resistance was not investigated.

RT is the main treatment for NPC [1]. As an effective and commonly used cancer treatment and the standard therapeutic intervention for various malignant tumors, IR causes DNA damage and destroys cancer cells to achieve its therapeutic effects [25]. Despite the high radiosensitivity of bulk cells, the existence of radioresistant cells in NPC tissue, however, lead to NPC recurrence and metastasis after radiation [26, 27]. In this study, we found that NPC cells with lower expression of miR-34c displayed less sensitivity to irradiation, which contribute to the radioresistance of NPC in clinic.

EMT is an important factor in promoting radioresistance in tumors [4]. In this study, we found that the lack of miR-34c in NPC cells elevated the expression of mesenchymal markers but decreased the epithelial markers, which indicated an anti-EMT role of miR-34c. Accordingly, miR-34c was found to inhibit the radioresistance in NPC cells in this study. Our previous studies have confirmed the relationship between EMT and radioresistance in NPC cell lines [8]. Both in vitro and in vivo experiments have shown that the radioresistant CNE-2R cell line undergoes EMT more readily than the CNE-2 NPC cell line. Furthermore, recent study has demonstrated that NPC cells with mesenchymal phenotypes displayed strong resistance to IR [28]. Therefore, whether the anti-radioresistant effect of miR-34c in the current study was mediated by the inhibition of EMT process remained to be studied in the future. It is reported that EMT is also strongly associated with cancer cell proliferation, invasion and metastasis [29]. We studied the role of miR-34c on these set of EMT-induced malignant behaviors in NPC cells and found that miR-34c inhibited the migration, invasion and proliferation of NPC. These anti-tumor effects in NPC was mediated by targeting β-catenin.

β-Catenin is a key molecule of a well-recognized cancer-promoting pathway, the Wnt/β-catenin signaling pathway, which promotes the development of tumors. The Wnt–β-catenin signaling pathway plays an important role in tumor EMT. It is reported that tumor cells acquire mesenchymal phenotype after increased β-catenin in cytoplasm translocating into nucleus and promoting transcription of EMT-related genes [28]. Moreover, in NPC and pancreatic cancer, elevated β-catenin was confirmed to be related to reduce radiosensitivity [30]. Therefore, to downregulate the expression level of β-catenin is an effective means to inhibit tumor progression and to restore sensitivity to radiotherapy. In breast cancer, β-catenin is reported to be targeted by miR-34c. However, this is the first study suggesting an inhibiting effect of miR-34c on β-catenin in NPCs. We found that miR-34c directly targeted the 3′-UTR region of β-catenin mRNA to decreased the expression level of β-catenin and thus lead to reduced EMT and radioresistance.

MSCs are heterogeneous progenitor cells that play an important role in tissue regeneration. As they can be isolated from various tissues and be readily cultured and engineered ex vivo [31], in addition to the tendency to specifically home in on tumor tissue [32], MSCs are ideal cells to deliver anti-tumor agents to tumor tissue [16, 33]. However, the large volume of MSCs undoubtedly places many restrictions on the direct application of MSCs in the treatment of cancer [34]. Unlike MSCs, exosomes are nanovesicles that are much easier for administration. As a result, MSC-derived exosomes may be an alternative method to deliver anti-tumor agents. Studies have shown that MSC-derived exosomes combined with RT can significantly reduce the growth, metastasis and radiation resistance of melanoma [18]. Based on this finding, we overexpressed miR-34c in MSCs and found an elevated expression level of miR-34c in MSC-derived exosomes (MSC-exo-miR-34c) accordingly. The MSC-exo-miR-34c deliver miR-34c to NPC cells and therefore inhibit EMT-induced malignant behaviours in NPCs. More importantly, MSC-exo-miR-34c could significantly ameliorate the resistance to radiotherapy in NPC cells. It is reported that IR could induce EMT [13, 35], which further generates radioresistance and reduces the efficiency of RT. The administration of MSC-exo-miR-34c could block the IR induced EMT and further restore the radiosensitivity in NPC cells.

In summary, we found for the first time that miR-34 is an important tumor suppressor-miR in NPCs that can significantly reduce EMT and radiation resistance. To utilize this anti-tumor effects of miR-34c, we modified MSCs as a “bio-factory” for exosomes overexpressing miR-34c. The administration of MSC-exo-miR-34c inhibit the malignant behaviors and promote the radiosensitivity in NPCs, which may act as an adjuvant treatment for IR.

## Conclusion

In conclusion, we demonstrate that miR-34c plays an important role in inhibiting the malignant behavior and radioresistance in NPCs, possibly by blocking the EMT process. β-Catenin was shown to be a direct target of miR-34c, and the anti-cancer effect of miR-34c is partly due to the inhibition of β-catenin expression. Exosomes derived from MSCs overexpressing miR-34c can be used to inhibit the development of tumors and promote the sensitivity of NPC to irradiation, thereby improving the therapeutic effect of radiation.

## Supplementary information

**Additional file 1: Figure S1.** Transfaction efficiency of MSC. (A) Percentage of GFP positive cells detect by flow cytometry. (B) PCR analysis of miR-34c levels in MSC. (A) (*P < 0.05; **P < 0.01; ***P < 0.001; ns: no statistical significance).

**Additional file 2: Figure S2.** Effect of miR-34c exosome on tumor without radiation in vivo. (A)CNE-2R cells were treated with PBS, and exosomes were injected subcutaneously into nude mice (n = 3 in each group). (B) Weight of tumor in each group. (C) Tumor volume in each group. (A)(*P < 0.05; **P < 0.01; ***P < 0.001; ns: no statistical significance).

**Additional file 3: Figure S3.** Images of HE staining of tumor samples. (A) miR-34c exosome group are more hollowed. (B) Tumor buddings.

## Data Availability

The datasets used and analyzed during the current study are available from the corresponding author on reasonable request.

## Abbreviations

3′ UTR: 3′untranslated region
EMT: epithelial-to-mesenchymal transition
miRNA: microRNA
GEO: Gene Expression Omnibus (GEO) database
NPC: nasopharyngeal carcinoma
RT: radiation therapy (RT)
miR-34c: microRNA-34c-5p
MSC: mesenchymal stem cells
CCK-8: Cell Counting Kit-8
L-Q model: linear quadratic model

## References

[1] Shield KD, Ferlay J, Jemal A, Sankaranarayanan R, Chaturvedi AK, Bray F, Soerjomataram I (2017). The global incidence of lip, oral cavity, and pharyngeal cancers by subsite in 2012. CA Cancer J Clin.

[2] Kwong D, Sham J, Choy D (1994). The effect of loco-regional control on distant metastatic dissemination in carcinoma of the nasopharynx: an analysis of 1301 patients. Int J Radiat Oncol Biol Phys.

[3] Fang FM, Tsai WL, Chien CY, Chen HC, Hsu HC, Huang TL, Lee TF, Huang HY, Lee CH (2010). Pretreatment quality of life as a predictor of distant metastasis and survival for patients with nasopharyngeal carcinoma. J Clin Oncol.

[4] Floor S, Van Staveren WC, Larsimont D, Dumont JE, Maenhaut C (2011). Cancer cells in epithelial-to-mesenchymal transition and tumor-propagating-cancer stem cells: distinct, overlapping or same populations. Oncogene.

[5] Tsai JH, Yang J (2013). Epithelial–mesenchymal plasticity in carcinoma metastasis. Genes Dev.

[6] Zhou P, Li B, Liu F, Zhang M, Wang Q, Liu Y, Yao Y, Li D (2017). The epithelial to mesenchymal transition (EMT) and cancer stem cells: implication for treatment resistance in pancreatic cancer. Mol Cancer.

[7] Nieto MA, Huang RY, Jackson RA, Thiery JP (2016). Emt: 2016. Cell.

[8] Lin G, Yu B, Liang Z, Li L, Qu S, Chen K, Zhou L, Lu Q, Sun Y, Zhu X (2018). Silencing of c-jun decreases cell migration, invasion, and EMT in radioresistant human nasopharyngeal carcinoma cell line CNE-2R. Onco Targets Ther.

[9] Okada N, Lin CP, Ribeiro MC, Biton A, Lai G, He X, Bu P, Vogel H, Jablons DM, Keller AC, Wilkinson JE (2014). A positive feedback between p53 and miR-34 miRNAs mediates tumor suppression. Genes Dev.

[10] Gu J, Wang G, Liu H, Xiong C (2018). SATB2 targeted by methylated miR-34c-5p suppresses proliferation and metastasis attenuating the epithelial-mesenchymal transition in colorectal cancer. Cell Prolif.

[11] Hagman Z, Haflidadottir BS, Ansari M, Persson M, Bjartell A, Edsjö A, Ceder Y (2013). The tumour suppressor miR-34c targets MET in prostate cancer cells. Br J Cancer.

[12] Russo V, Paciocco A, Affinito A, Roscigno G, Fiore D, Palma F, Galasso M, Volinia S, Fiorelli A, Esposito CL, Nuzzo S (2018). Aptamer-miR-34c conjugate affects cell proliferation of non-small-cell lung cancer cells. Molecular therapy. Nucleic Acids.

[13] Marie-Egyptienne DT, Lohse I, Hill RP (2013). Cancer stem cells, the epithelial to mesenchymal transition (EMT) and radioresistance: potential role of hypoxia. Cancer Lett.

[14] Kourembanas S (2015). Exosomes: vehicles of intercellular signaling, biomarkers, and vectors of cell therapy. Annu Rev Physiol.

[15] Lou G, Song X, Yang F, Wu S, Wang J, Chen Z, Liu Y (2015). Exosomes derived from miR-122-modified adipose tissue-derived MSCs increase chemosensitivity of hepatocellular carcinoma. J Hematol Oncol.

[16] Lang FM, Hossain A, Gumin J, Momin EN, Shimizu Y, Ledbetter D, Shahar T, Yamashita S, Parker Kerrigan B, Fueyo J, Sawaya R (2018). Mesenchymal stem cells as natural biofactories for exosomes carrying miR-124a in the treatment of gliomas. Neuro-oncology.

[17] Gatti S, Bruno S, Deregibus MC, Sordi A, Cantaluppi V, Tetta C, Camussi G (2011). Microvesicles derived from human adult mesenchymal stem cells protect against ischaemia-reperfusion-induced acute and chronic kidney injury. Nephrol Dialysis Transpl.

[18] de Farias V, O’Valle F, Serrano-Saenz S, Anderson P, Andrés E, López-Peñalver J, Tovar I, Nieto A, Santos A, Martín F, Expósito J (2018). Exosomes derived from mesenchymal stem cells enhance radiotherapy-induced cell death in tumor and metastatic tumor foci. Mol Cancer.

[19] Figueroa J, Phillips LM, Shahar T, Hossain A, Gumin J, Kim H, Bean AJ, Calin GA, Fueyo J, Walters ET, Kalluri R (2017). Exosomes from glioma-associated mesenchymal stem cells increase the tumorigenicity of glioma stem-like cells via transfer of miR-1587. Cancer Res.

[20] Lewis BP, Burge CB, Bartel DP (2005). Conserved seed pairing, often flanked by adenosines, indicates that thousands of human genes are microRNA targets. Cell.

[21] Wang X (2008). miRDB: a microRNA target prediction and functional annotation database with a wiki interface. RNA.

[22] Wei CY, Zhu MX, Yang YW, Zhang PF, Yang X, Peng R, Gao C, Lu JC, Wang L, Deng XY, Lu NH (2019). Downregulation of RNF128 activates Wnt/β-catenin signaling to induce cellular EMT and stemness via CD44 and CTTN ubiquitination in melanoma. J Hematol Oncol.

[23] Li YQ, Ren XY, He QM, Xu YF, Tang XR, Sun Y, Zeng MS, Kang TB, Liu N, Ma J (2015). miR-34c suppresses tumor growth and metastasis in nasopharyngeal carcinoma by targeting MET. Cell Death & Disease.

[24] Yang S, Li Y, Gao J, Zhang T, Li S, Luo A, Chen H, Ding F, Wang X, Liu Z (2013). MicroRNA-34 suppresses breast cancer invasion and metastasis by directly targeting Fra-1. Oncogene.

[25] Surova O, Zhivotovsky B (2013). Various modes of cell death induced by DNA damage. Oncogene.

[26] Luftig M (2013). Heavy LIFting: tumor promotion and radioresistance in NPC. J Clin Investig.

[27] Zhao L, Fong AH, Liu N, Cho WC (2018). Molecular subtyping of nasopharyngeal carcinoma (NPC) and a microRNA-based prognostic model for distant metastasis. J Biomed Sci.

[28] Luo M, Wu C, Guo E, Peng S, Zhang L, Sun W, Liu D, Hu G, Hu G (2019). FOXO3a knockdown promotes radioresistance in nasopharyngeal carcinoma by inducing epithelial-mesenchymal transition and the Wnt/β-catenin signaling pathway. Cancer Lett.

[29] Kalluri R, Weinberg RA (2009). The basics of epithelial-mesenchymal transition. J Clin Investig.

[30] Xue J, Zhu W, Song J, Jiao Y, Luo J, Yu C, Zhou J, Wu J, Chen M, Ding WQ, Cao J (2018). Activation of PPARα by clofibrate sensitizes pancreatic cancer cells to radiation through the Wnt/β-catenin pathway. Oncogene.

[31] Uccelli A, Moretta L, Pistoia V (2008). Mesenchymal stem cells in health and disease. Nat Rev Immunol.

[32] Kidd S, Spaeth E, Dembinski JL, Dietrich M, Watson K, Klopp A, Battula VL, Weil M, Andreeff M, Marini FC (2009). Direct evidence of mesenchymal stem cell tropism for tumor and wounding microenvironments using in vivo bioluminescent imaging. Stem Cells (Dayton, Ohio).

[33] Rossignoli F, Spano C, Grisendi G, Foppiani EM, Golinelli G, Mastrolia I, Bestagno M, Candini O, Petrachi T, Recchia A, Miselli F (2019). MSC-Delivered Soluble TRAIL and paclitaxel as novel combinatory treatment for pancreatic adenocarcinoma. Theranostics.

[34] Fischer UM, Harting MT, Jimenez F, Monzon-Posadas WO, Xue H, Savitz SI, Laine GA, Cox CS (2009). Pulmonary passage is a major obstacle for intravenous stem cell delivery: the pulmonary first-pass effect. Stem Cells Dev.

[35] Dave B, Mittal V, Tan NM, Chang JC (2012). Epithelial-mesenchymal transition, cancer stem cells and treatment resistance. Breast Cancer Res.

