# Supplementary figures and images for: Exosomes overexpressing miR-34c inhibit malignant behavior and reverse the radioresistance of nasopharyngeal carcinoma
Source: J Transl Med. 2020 Jan 8;18:12. doi: 10.1186/s12967-019-02203-z (PMC6947927; doi:10.1186/s12967-019-02203-z)

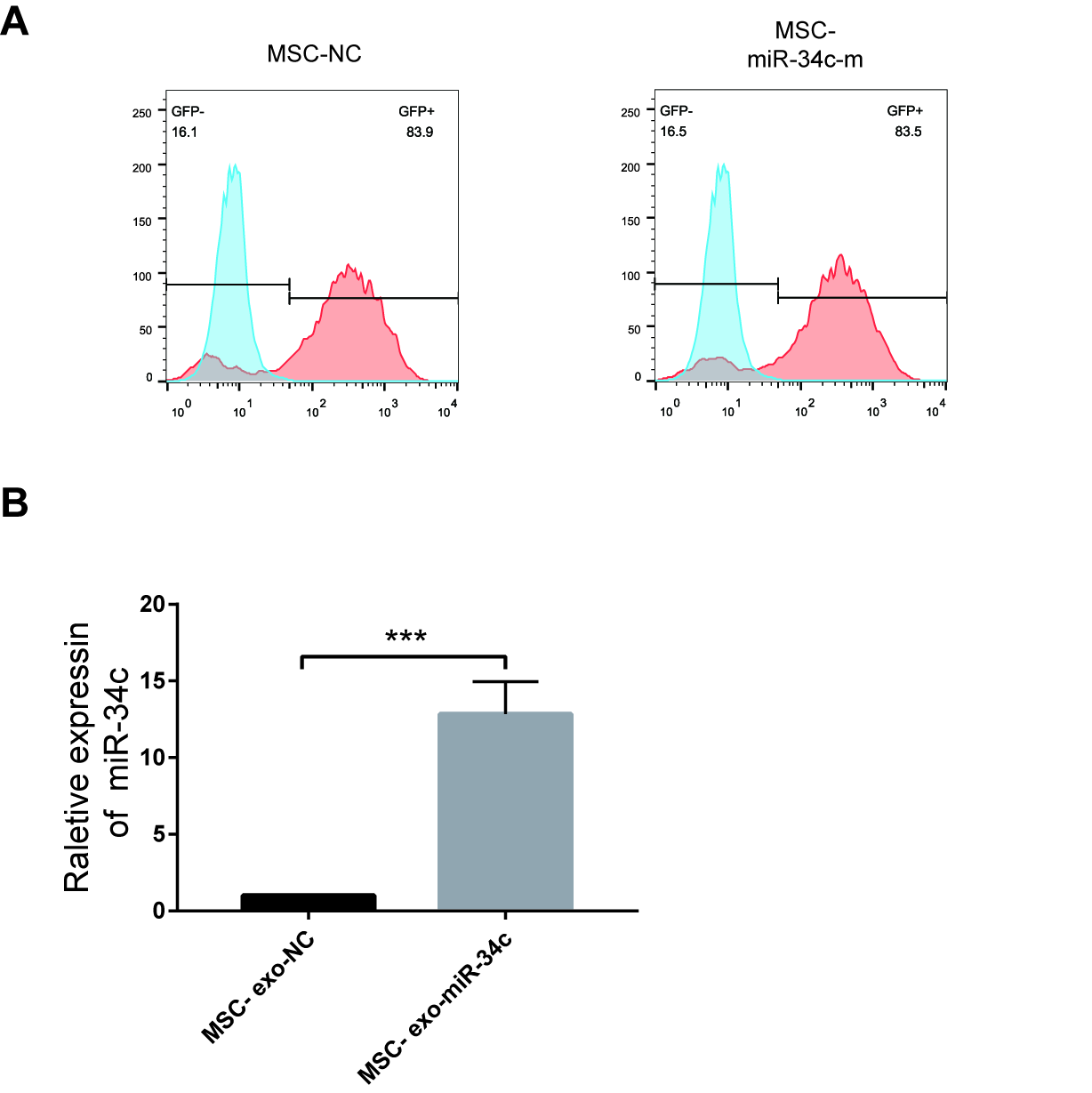

Supplement: Supplementary file 1 — Additional file 1: Figure S1. Transfaction efficiency of MSC. (A) Percentage of GFP positive cells detect by flow cytometry. (B) PCR analysis of miR-34c levels in MSC. (A) (*P < 0.05; **P < 0.01; ***P < 0.001; ns: no statistical significance). [file 12967_2019_2203_MOESM1_ESM.tif]

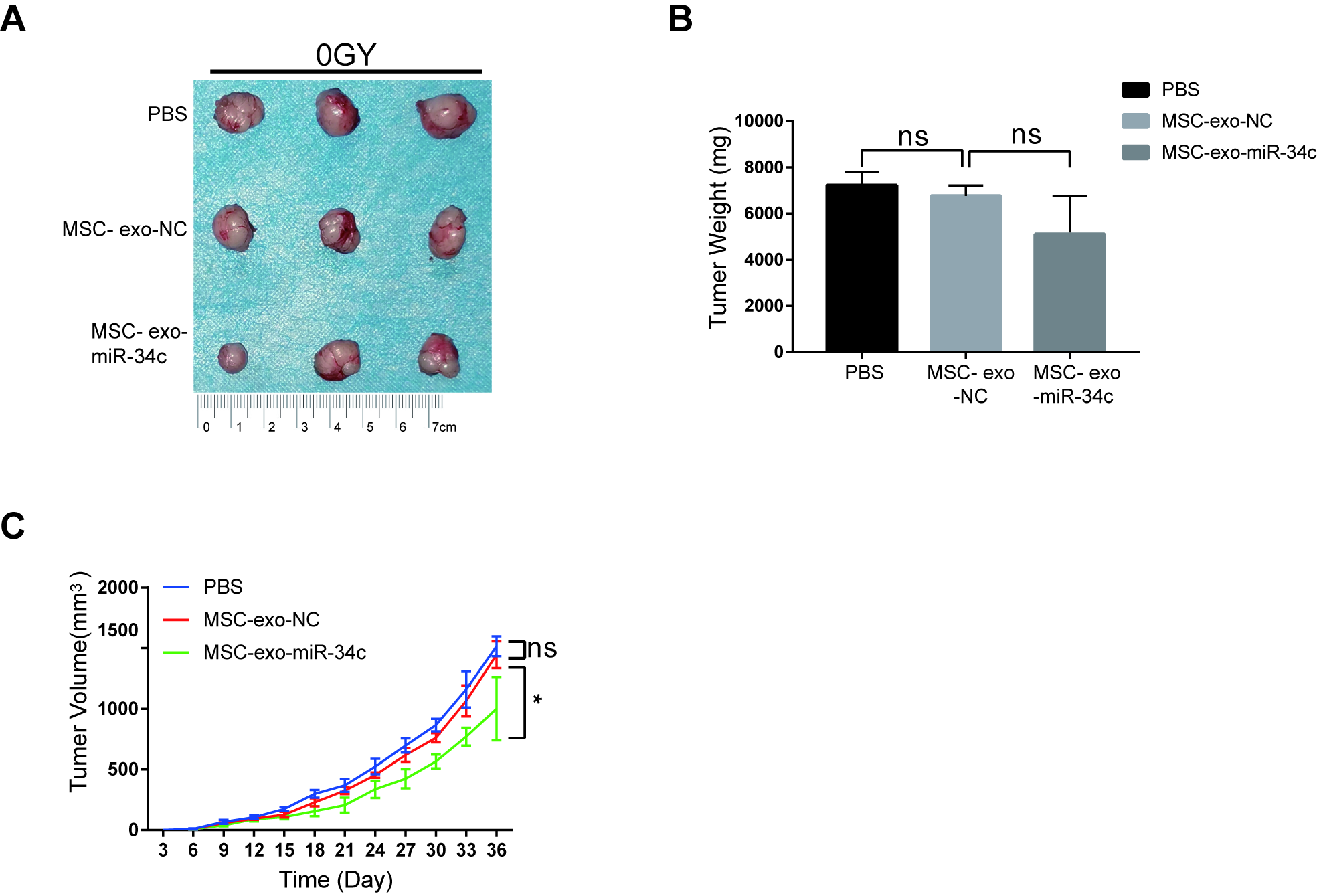

Supplement: Supplementary file 2 — Additional file 2: Figure S2. Effect of miR-34c exosome on tumor without radiation in vivo. (A)CNE-2R cells were treated with PBS, and exosomes were injected subcutaneously into nude mice (n = 3 in each group). (B) Weight of tumor in each group. (C) Tumor volume in each group. (A)(*P < 0.05; **P < 0.01; ***P < 0.001; ns: no statistical significance). [file 12967_2019_2203_MOESM2_ESM.tif]

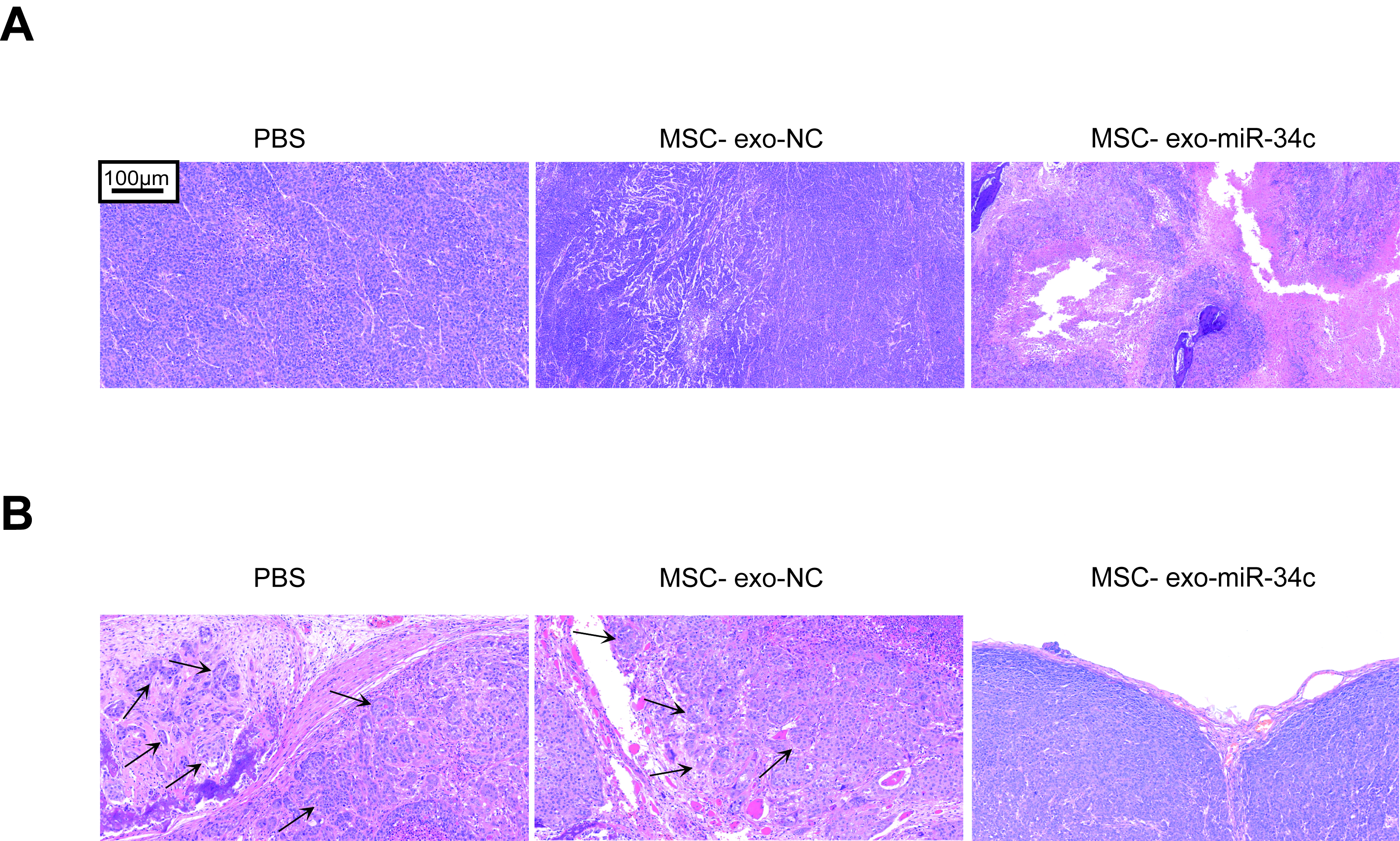

Supplement: Supplementary file 3 — Additional file 3: Figure S3. Images of HE staining of tumor samples. (A) miR-34c exosome group are more hollowed. (B) Tumor buddings. [file 12967_2019_2203_MOESM3_ESM.tif]
